# Supplementary material for: Content-rich biological network constructed by mining PubMed abstracts
Source: BMC Bioinformatics. 2004 Oct 8;5:147. doi: 10.1186/1471-2105-5-147 (PMC528731; doi:10.1186/1471-2105-5-147)
Supplement: Additional File 2 — The original results of the above study (non-essential files are deleted to keep the file size under the limit set by BMC bioinformatics). [file 1471-2105-5-147-S2.bz2 › chilibotAdditionalFile2/dip10/427ID11470772E10357/html/CCL2_CCR2.html]

 


 **CCL2** and **CCR2** 
  
Found 2851 abstracts in PubMed, retrieved 10.  
 

 What does Google say? 
 PDF only 
| .edu only 

---

**Interactive relationship** (e.g. stimulation, inhibition, etc)

**Non-interactive relationship** (e.g. studied together, co-existance, homology, etc.)

- The protein level of MCP 1  [ **CCR2** ]   [ **CCL2** ]  in the mild hypothermic subgroups was 8.7 7.6 ng.g tissue 1 6 h after reperfusion P <.  Ref: 12887739 Zhonghua Yi Xue Za Zhi, 2003
- Although no associations were found in alleles or genotypes, MCP 1  [ **CCR2** ]   [ **CCL2** ]  2518A G genotype affected the age at onset of PD patients.  Ref: 12889089 Mov Disord, 2003
- CONCLUSIONS Both MCP 1  [ **CCR2** ]   [ **CCL2** ]  and the M CSF c fms system are involved in the atherogenesis of de novo lesions.  Ref: 12892039 J Cardiol, 2003
- Neutralizing antibodies against human RANTES, MIP 1alpha, MIP 1beta, alpha interferon IFN alpha, IFN beta, IFN gamma, interleukin 4 IL 4, IL 10, IL 13, IL 16, MCP 1  [ **CCR2** ]   [ **CCL2** ] , MCP  [ **CCL2** ]  3, tumor necrosis factor alpha TNF alpha, or TNF beta failed to reverse the HIV 1 suppressive activity.  Ref: 12885891 J VirolJ Virol, 2003
- The MCP 1  [ **CCR2** ]   [ **CCL2** ]  protein level in the cortical ischemic cores of the normothermic subgroups began to increase since the 6th hour afer reperfusion 22.5 8.7 ng.g tissue 7 times that in the sham operation samples, P <.  Ref: 12887739 Zhonghua Yi Xue Za Zhi, 2003
- The possible connection between periodontitis and atherosclerosis through IL 8 and MCP 1  [ **CCR2** ]   [ **CCL2** ]  is discussed.  Ref: 12887346 J Clin PeriodontolJ Clin Periodontol, 2003
- OBJECTIVE To investigate the effect of intraischemic mild hypothermia on the protein levels of interleukin IL 1beta and monocyte chemoattractant protein MCP 1  [ **CCR2** ]   [ **CCL2** ]  in the ischemic core of rat cortex after transient focal cerebralischemia.  Ref: 12887739 Zhonghua Yi Xue Za Zhi, 2003
- Whereas treatment with infectious virus induced a rapid down regulation of the  **CCL2**  monocyte chemoattractant protein 1  [ **CCL2** ]  MCP 1  [ **CCR2** ]   [ **CCL2** ]  specific receptor  **CCR2** , inactivated virus did not significantly alter  **CCR2**  surface expression.  Ref: 12885942 J Leukoc Biol, 2003
- CONCLUSION Mild hypothermia reduces the level of MCP 1  [ **CCR2** ]   [ **CCL2** ]  in the cortex after cerebral ischemia reperfusion which may be one of the important mechanisms of the neuroprotective effects of mild hypothermia.  Ref: 12887739 Zhonghua Yi Xue Za Zhi, 2003
- We studied polymorphisms in the genes for monocyte chemoattractant protein 1  [ **CCL2** ]  MCP 1  [ **CCR2** ]   [ **CCL2** ]  and CC chemokine receptor CCR 2  [ **CCR2** ]  in 171 Parkinson s disease PD patients and 340 controls.  Ref: 12889089 Mov Disord, 2003
- The expression of MCP 1  [ **CCR2** ]   [ **CCL2** ]  positive cells almost coincided with the macrophages.  Ref: 12892039 J Cardiol, 2003
- ... IL 6, MIP 2 and MCP 1  [ **CCR2** ]   [ **CCL2** ]  were increased by 4 8 hours.  Ref: 12883092 Toxicol SciToxicol Sci, 2003
- In contrast, staining for MCP 1  [ **CCR2** ]   [ **CCL2** ]  was little seen in the stellate vascular smooth muscle cells.  Ref: 12892039 J Cardiol, 2003
- This effect was also detected in a second PD group, suggesting a possible involvement of MCP 1  [ **CCR2** ]   [ **CCL2** ]  in PD.  Ref: 12889089 Mov Disord, 2003
- The protein levels of IL 1beta and MCP 1  [ **CCR2** ]   [ **CCL2** ]  in the cortical ischemic core were measured by ELISA.  Ref: 12887739 Zhonghua Yi Xue Za Zhi, 2003
- In parallel, the response to  **CCL2**  MCP 1  [ **CCR2** ]   [ **CCL2** ]  was lost after infection with active virus Neither a  **CCL2**  MCP 1  [ **CCR2** ]   [ **CCL2** ]  induced shift of intracellular calcium concentrations nor the chemotactic response to  **CCL2**  MCP 1  [ **CCR2** ]   [ **CCL2** ]  was detectable.  Ref: 12885942 J Leukoc Biol, 2003
- Monocyte chemoattractant protein MCP 1  [ **CCR2** ]   [ **CCL2** ]  and macrophage colony stimulating factor M CSF are two of the associated chemokines, but their role in coronary artery disease has not been sufficiently clarified.  Ref: 12892039 J Cardiol, 2003
- This study investigated the expression of MCP 1  [ **CCR2** ]   [ **CCL2** ]  and c fms M CSF receptor in human coronary tissues.  Ref: 12892039 J Cardiol, 2003
- The release of the chemokines interleukin IL 8 and macrophage chemoattractant protein MCP 1  [ **CCR2** ]   [ **CCL2** ]  in the cultures decreased twofold over time.  Ref: 12887346 J Clin PeriodontolJ Clin Periodontol, 2003
- IL 6, MIP 2 and MCP 1  [ **CCR2** ]   [ **CCL2** ]  appear to follow the toxicity.  Ref: 12883092 Toxicol SciToxicol Sci, 2003
- These results demonstrate that homocysteine can trigger overexpression of the MCP 1  [ **CCR2** ]   [ **CCL2** ]  gene by altering the intracellular redox status, suggesting that the homocysteine induced changes in the intracellular redox status play an important role in modulating the expression of MCP  [ **CCL2** ]  l in endothelial cells.  Ref: 12883331 J Cardiovasc Pharmacol, 2003
- In a mouse macrophage like cell line, the same beta isoform activation increases the release of the MCP 1  [ **CCR2** ]   [ **CCL2** ]  chemokine.  Ref: 12892998 Mol Aspects MedMol Aspects Med, 2003
- However, the M CSF c fms system, rather than MCP 1  [ **CCR2** ]   [ **CCL2** ] , is more important in the late stage of restenosis.  Ref: 12892039 J Cardiol, 2003
- The following primary antibodies were used anti MCP 1  [ **CCR2** ]   [ **CCL2** ] , anti c fms, anti macrophages and anti alpha smooth muscle actin.  Ref: 12892039 J Cardiol, 2003
- Expression patterns of MCP 1  [ **CCR2** ]   [ **CCL2** ]  and c fms exhibited no difference between the two lesion types.  Ref: 12892039 J Cardiol, 2003
- CONCLUSION On the basis of previous studies and the present case, the high production of IL 8 and MCP 1  [ **CCR2** ]   [ **CCL2** ]  by monocytes in LPS stimulated WBCC from periodontitis patients is most likely acquired, as their levels decrease over time when the periodontal infection is controlled.  Ref: 12887346 J Clin PeriodontolJ Clin Periodontol, 2003
- Homocysteine stimulated MCP 1  [ **CCR2** ]   [ **CCL2** ]  mRNA expression and protein production in a time dependent and dose dependent manner in endothelial cells, decreased intracellular glutathione GSH and protein thiol levels, as well as G6PDH activity and NADPH levels.  Ref: 12883331 J Cardiovasc Pharmacol, 2003
